# Supplementary material for: Assessment of the association between body composition and risk of non-alcoholic fatty liver
Source: PLoS One. 2021 Apr 1;16(4):e0249223. doi: 10.1371/journal.pone.0249223 (PMC8016222; doi:10.1371/journal.pone.0249223)
Supplement: S1 Table — (DOCX) [file pone.0249223.s001.docx]

S1 Table. Demographic and basic information of the male participants in the group without NAFLD and NAFLD patients

|  | The group without NAFLD (N=1415) | | NAFLD patient  (N=745) | | P-value^*^ | OR | 95% C.I. | |
| --- | --- | --- | --- | --- | --- | --- | --- | --- |
|  | Mean | SD | Mean | SD |  |  | Lower | Upper |
| Age (years) | 35 | 9 | 47 | 8 | 0.000 | 1.171 | 1.148 | 1.194 |
| Weight (Kg) | 80.33 | 11.92 | 93.12 | 11.90 | 0.000 | 1.087 | 1.074 | 1.101 |
| BMI (Kg/m²) | 25.29 | 4.24 | 29.77 | 3.40 | 0.000 | 1.322 | 1.271 | 1.375 |
| WC (cm) | 95.35 | 14.51 | 112.26 | 11.50 | 0.000 | 1.095 | 1.082 | 1.109 |
| Physical Activity (MET/24h) | 25.7 | 5.4 | 19.9 | 4.9 | 0.000 | 0.813 | 0.789 | 0.837 |
| Energy (Kcal) | 2377.71 | 413.00 | 2671.32 | 442.70 | 0.000 | 1.002 | 1.001 | 1.002 |
| FBS (mg/dL) | 99.35 | 13.76 | 116.55 | 11.97 | 0.000 | 1.097 | 1.084 | 1.111 |
| LDL-C (mg/dL) | 94.70 | 16.14 | 112.23 | 17.77 | 0.000 | 1.061 | 1.052 | 1.071 |
| HDL-C (mg/dL) | 46.74 | 7.99 | 38.33 | 6.73 | 0.000 | 0.866 | 0.848 | 0.884 |
| TG (mg/dL) | 182.59 | 25.93 | 207.77 | 26.26 | 0.000 | 1.036 | 1.031 | 1.042 |
| TC(mg/dL) | 176.26 | 18.70 | 195.29 | 16.04 | 0.000 | 1.064 | 1.054 | 1.073 |
| SBP (mmHg) | 12.35 | 1.65 | 12.76 | 1.61 | 0.000 | 1.164 | 1.077 | 1.259 |
| DBP (mmHg) | 8.05 | 1.07 | 8.69 | 0.98 | 0.000 | 1.790 | 1.571 | 2.039 |
| GGT (mg/dL) | 24.66 | 10.92 | 37.61 | 13.55 | 0.000 | 1.088 | 1.075 | 1.101 |
| ALT (UL/L) | 38.48 | 14.04 | 57.12 | 14.00 | 0.000 | 1.092 | 1.080 | 1.105 |
| AST (UL/L) | 31.22 | 13.53 | 46.68 | 11.91 | 0.000 | 1.092 | 1.079 | 1.105 |
| LSM (dB) | 212.15 | 42.80 | 272.64 | 35.78 | 0.000 | 1.036 | 1.031 | 1.040 |
| Left Arm Fat (kg) | 2580.68 | 1703.02 | 4076.46 | 1521.10 | 0.000 | 1.672 | 1.538 | 1.817 |
| Right Arm Fat (kg) | 2650.41 | 1727.77 | 4319.08 | 1661.78 | 0.000 | 1.704 | 1.569 | 1.850 |
| Left Leg Fat (kg) | 3526.72 | 2498.03 | 6551.14 | 2480.77 | 0.000 | 1.542 | 1.455 | 1.634 |
| Right Leg Fat (kg) | 3533.20 | 2565.80 | 6429.73 | 2449.66 | 0.000 | 1.508 | 1.425 | 1.596 |
| Abdominal Fat (kg) | 6247.55 | 3907.61 | 11683.11 | 3234.83 | 0.000 | 1.444 | 1.380 | 1.512 |
| Total Fat (kg) | 21464.29 | 9474.80 | 36160.63 | 7359.05 | 0.000 | 1.183 | 1.159 | 1.207 |
| Left Arm FatFree (kg) | 1868.99 | 473.44 | 1862.47 | 410.63 | 0.825 | 0.969 | 0.732 | 1.282 |
| Right Arm FatFree (kg) | 1858.63 | 458.09 | 1891.00 | 413.04 | 0.262 | 1.178 | 0.885 | 1.568 |
| Left Leg FatFree (kg) | 4589.86 | 639.69 | 4581.01 | 658.05 | 0.833 | 0.979 | 0.804 | 1.192 |
| Right Leg FatFree (kg) | 4614.46 | 645.41 | 4563.02 | 594.92 | 0.209 | 0.787 | 0.717 | 1.075 |
| Abdominal FatFree (kg) | 11749.67 | 1337.13 | 11280.94 | 1118.51 | 0.000 | 0.748 | 0.675 | 0.829 |
| Total FatFree (kg) | 24067.34 | 2996.03 | 23709.76 | 2451.78 | 0.052 | 0.956 | 0.913 | 1.001 |

BMI, body mass index; FBS, Fasting Blood Sugar; HDL-C, high-density lipoprotein cholesterol; LDL-C, low-density lipoprotein cholesterol; TG, Triglyceride; TC, Total cholesterol; SBP, Systolic Blood Pressure; DBP, Diastolic Blood Pressure; GGT, gamma-glutamyl transferase; ALT, alanine aminotransferase; AST, aspartate aminotransferase; LSM, liver stiffness measurement by FibroScan;

*. Independent samples t-tests
