# Supplementary material for: Assessment of the association between body composition and risk of non-alcoholic fatty liver
Source: PLoS One. 2021 Apr 1;16(4):e0249223. doi: 10.1371/journal.pone.0249223 (PMC8016222; doi:10.1371/journal.pone.0249223)
Supplement: S2 Table — (DOCX) [file pone.0249223.s002.docx]

S2 Table. Demographic and basic information of the female participants in the group without NAFLD and NAFLD patients

|  | The group without NAFLD (N=1415) | | NAFLD patient  (N=745) | | P-value^*^ | OR | 95% C.I. | |
| --- | --- | --- | --- | --- | --- | --- | --- | --- |
|  | Mean | SD | Mean | SD |  |  | Lower | Upper |
| Age (years) | 36 | 10 | 49 | 8 | 0.000 | 1.144 | 1.125 | 1.163 |
| Weight (Kg) | 82.24 | 13.31 | 97.65 | 10.96 | 0.000 | 1.098 | 1.084 | 1.112 |
| BMI (Kg/m²) | 26.08 | 4.29 | 31.16 | 3.87 | 0.000 | 1.316 | 1.269 | 1.365 |
| WC (cm) | 98.87 | 18.53 | 121.53 | 12.56 | 0.000 | 1.085 | 1.074 | 1.096 |
| Physical Activity (MET/24h) | 23.4 | 4.8 | 17.9 | 4.3 | 0.000 | 0.790 | 0.765 | 0.816 |
| Energy (Kcal) | 2422.80 | 408.29 | 2684.95 | 468.85 | 0.000 | 1.001 | 1.001 | 1.002 |
| FBS (mg/dL) | 100.23 | 14.68 | 118.55 | 13.35 | 0.000 | 1.086 | 1.075 | 1.098 |
| LDL-C (mg/dL) | 94.78 | 16.41 | 111.48 | 17.18 | 0.000 | 1.059 | 1.050 | 1.068 |
| HDL-C (mg/dL) | 49.11 | 8.24 | 40.52 | 7.70 | 0.000 | 0.881 | 0.865 | 0.897 |
| TG (mg/dL) | 193.22 | 26.65 | 216.63 | 24.39 | 0.000 | 1.035 | 1.029 | 1.041 |
| TC(mg/dL) | 177.10 | 16.65 | 193.30 | 16.29 | 0.000 | 1.060 | 1.050 | 1.069 |
| SBP (mmHg) | 12.66 | 1.65 | 13.29 | 1.58 | 0.000 | 1.270 | 1.174 | 1.373 |
| DBP (mmHg) | 8.08 | 0.99 | 8.89 | 0.96 | 0.000 | 2.268 | 1.968 | 2.613 |
| GGT (mg/dL) | 23.61 | 10.21 | 34.36 | 12.80 | 0.000 | 1.084 | 1.070 | 1.098 |
| ALT (UL/L) | 42.94 | 14.78 | 58.63 | 13.44 | 0.000 | 1.075 | 1.064 | 1.087 |
| AST (UL/L) | 33.99 | 14.46 | 51.66 | 12.60 | 0.000 | 1.091 | 1.079 | 1.103 |
| LSM (dB) | 220.60 | 43.44 | 274.93 | 34.02 | 0.000 | 1.032 | 1.028 | 1.037 |
| Left Arm Fat (kg) | 2567.27 | 1569.30 | 4045.54 | 1614.75 | 0.000 | 1.740 | 1.596 | 1.896 |
| Right Arm Fat (kg) | 2624.79 | 1682.88 | 4088.64 | 1558.15 | 0.000 | 1.675 | 1.542 | 1.819 |
| Left Leg Fat (kg) | 4109.54 | 2633.76 | 6726.62 | 2280.27 | 0.000 | 1.481 | 1.399 | 1.569 |
| Right Leg Fat (kg) | 4136.19 | 2665.93 | 6891.93 | 2328.47 | 0.000 | 1.494 | 1.411 | 1.583 |
| Abdominal Fat (kg) | 6696.58 | 4472.14 | 12410.97 | 3634.28 | 0.000 | 1.339 | 1.291 | 1.388 |
| Total Fat (kg) | 23279.60 | 9715.82 | 37766.47 | 8179.68 | 0.000 | 1.164 | 1.143 | 1.186 |
| Left Arm FatFree (kg) | 1831.41 | 462.26 | 1762.49 | 432.28 | 0.016 | 0.711 | 0.538 | 0.940 |
| Right Arm FatFree (kg) | 1867.11 | 463.02 | 1758.11 | 422.19 | 0.000 | 0.577 | 0.434 | 0.767 |
| Left Leg FatFree (kg) | 4534.16 | 617.62 | 4441.45 | 662.80 | 0.022 | 0.794 | 0.652 | 0.967 |
| Right Leg FatFree (kg) | 4578.80 | 612.00 | 4453.02 | 655.17 | 0.002 | 0.727 | 0.595 | 0.888 |
| Abdominal FatFree (kg) | 11615.02 | 1327.85 | 10943.48 | 1107.48 | 0.000 | 0.646 | 0.580 | 0.719 |
| Total FatFree (kg) | 23911.37 | 2987.52 | 22869.65 | 2472.96 | 0.000 | 0.871 | 0.830 | 0.914 |

BMI, body mass index; FBS, Fasting Blood Sugar; HDL-C, high-density lipoprotein cholesterol; LDL-C, low-density lipoprotein cholesterol; TG, Triglyceride; TC, Total cholesterol; SBP, Systolic Blood Pressure; DBP, Diastolic Blood Pressure; GGT, gamma-glutamyl transferase; ALT, alanine aminotransferase; AST, aspartate aminotransferase; LSM, liver stiffness measurement by FibroScan;

*. Independent samples t-tests
