# Supplementary material for: Assessment of the association between body composition and risk of non-alcoholic fatty liver
Source: PLoS One. 2021 Apr 1;16(4):e0249223. doi: 10.1371/journal.pone.0249223 (PMC8016222; doi:10.1371/journal.pone.0249223)
Supplement: Attachment — Submitted filename: Response to Reviewers.docx [file pone.0249223.s004.docx]

**Response to reviewers**

**Dear Editor and Reviewers**

We highly appreciate for your detailed and valuable comments on our manuscript. The suggestions were quite helpful for us and we incorporated them in the revised paper. We had referred to literatures and papers and reconstructed the paper to improve the quality of our manuscript. It is important to note that almost all recommendations were corrected and accordingly, we hope these changes could be acceptable to dear editor and respectful reviewers. Our responses to your comments are as follow:

Reviewer 1

1. The manuscript should be proofread by professional editing service.

- The manuscript was reviewed by two experts and we try to correct all grammatical errors. Also certificate for correcting grammatical mistake have sent with manuscript.

2. Table 2 is confused. The authors may consider separating the results into 2 tables including one in males and another one in females.

- Because of the beneficial recommendation of reviewers, table 2 reanalysed with the sex-separated and this analysis was performed in tables 2 and 3.

3. Based on your interpretation, the female would have a high risk of developing NAFLD, if they have total fat and abdominal fat more than 32.23% and 21.42%, respectively. Male would have a high risk of developing NAFLD, if they have total fat and abdominal fat more than 26.73% and 13.76%, respectively. It seems males have a high incidence of developing NAFLD since they require low content of body fat composition to develop NAFLD. In animal studies, it is common that scientists use male mice to study NAFLD because male mice develop NAFLD easily. Could you discuss this aspect in detail in your discussion?

- While thanking the reviewers for pointing out this important point, the following sentence was added to the discussion: "Notably, percentage of total fat in women and men of the same age is remarkably different. In fact, total fat is divided into “essential” and “storage" fats. Essential fat has been defined as the fundamental fat for normal physiologic functioning which consists of around 3% and 12% total body of males and females, respectively. On the other hand, storage fat is considered an energy reserve which is expendable and stored in adipose tissue. Thus, the average total fat of an individual (essential fat plus storage fat) in men and women is estimated to be 18-24 percent and 25-31 percent, respectively. Since the percentage of total fat in women is higher than men due to their physiological condition, the amount of total fat affected in diseases such as NAFLD should typically be higher in them than in males. Similarly, in men, the lower amount of total and abdominal fat affects the development of fatty liver, which does not necessarily mean that they develop NAFLD easily."

4. Did you check inflammatory cytokines or blood cell contents in your study? It will be interesting to validate if NAFLD patients have a low level of inflammation since adipokines have been linked to inflammation.

- We so appreciate for pointing out this point, but in the present study, these tests were not performed due to financial constraints. But this issue was mentioned in the limitations for future studies.

Reviewer 2

λ    In this study, Ariya et al. seek the association between body composition with risk of NAFLD. The design and procedures of this study sound reasonable and I have no major comments or criticisms for this manuscript. It would be easier for readers to understand if the authors show ROC curves in figures, not Table 3, with p values. Table 2 is confusing and misleading. It looks like the authors show correlation between male and female (e.g., correlation between male left arm fat and female right arm fat), which does not make sense. I think data Table 1 show combined data of male and female, and the authors want to show data for both genders separately. If so, Table 1 is enough to show without Table 2, and if the authors really need to show male and female data separately, format of tables should be same as Table 1, not combined like current Table 2.

- We appreciate your valuable comments. Table 3 (now shown as Table 4 in the manuscript) and Figure 1 are reported separately, so it was not possible to display all variables such as "Cut-off Point" in Figure 1. Again, if the reviewer's opinion is to delete this table, we will sincerely delete this table. Besides, it is noteworthy mentioning because of the beneficial recommendation of reviewers, table 2 reanalyzed with the sex-separated and this analysis was performed in tables 2 and 3.
