# Supplementary material for: Assessment of the association between body composition and risk of non-alcoholic fatty liver
Source: PLoS One. 2021 Apr 1;16(4):e0249223. doi: 10.1371/journal.pone.0249223 (PMC8016222; doi:10.1371/journal.pone.0249223)
Supplement: Attachment — Submitted filename: Response to Reviewers 2.docx [file pone.0249223.s005.docx]

**Response to reviewers**

**Dear Editor and Reviewers**

On behalf of all the authors of the article, I would like to thank the respected editors and reviewers who considered our article worthy of publication in the prestigious journal of Plos ONE and made suggestions to improve the article. We did our best to correct the recommendations and we hope that the article in its current form will acceptable to dear editor and the respected reviewers.

**Comments to the Author**

1. If the authors have adequately addressed your comments raised in a previous round of review and you feel that this manuscript is now acceptable for publication, you may indicate that here to bypass the “Comments to the Author” section, enter your conflict of interest statement in the “Confidential to Editor” section, and submit your "Accept" recommendation.

Reviewer #1: (No Response)

Reviewer #2: All comments have been addressed

2. Is the manuscript technically sound, and do the data support the conclusions?

The manuscript must describe a technically sound piece of scientific research with data that supports the conclusions. Experiments must have been conducted rigorously, with appropriate controls, replication, and sample sizes. The conclusions must be drawn appropriately based on the data presented.

Reviewer #1: Yes

Reviewer #2: Yes

3. Has the statistical analysis been performed appropriately and rigorously?

Reviewer #1: Yes

Reviewer #2: Yes

4. Have the authors made all data underlying the findings in their manuscript fully available?

The [PLOS Data policy](http://www.plosone.org/static/policies.action#sharing) requires authors to make all data underlying the findings described in their manuscript fully available without restriction, with rare exception (please refer to the Data Availability Statement in the manuscript PDF file). The data should be provided as part of the manuscript or its supporting information, or deposited to a public repository. For example, in addition to summary statistics, the data points behind means, medians and variance measures should be available. If there are restrictions on publicly sharing data—e.g. participant privacy or use of data from a third party—those must be specified.

Reviewer #1: Yes

Reviewer #2: Yes

5. Is the manuscript presented in an intelligible fashion and written in standard English?

PLOS ONE does not copyedit accepted manuscripts, so the language in submitted articles must be clear, correct, and unambiguous. Any typographical or grammatical errors should be corrected at revision, so please note any specific errors here.

Reviewer #1: Yes

Reviewer #2: Yes

6. Review Comments to the Author

Please use the space provided to explain your answers to the questions above. You may also include additional comments for the author, including concerns about dual publication, research ethics, or publication ethics. (Please upload your review as an attachment if it exceeds 20,000 characters)

Reviewer #1: As gender difference has been discussed in NAFLD development, the authors may want to include two tables showing data as the table 1 displayed.

Reviewer #2: In this study, Ariya et al. seek the association between body composition with risk of NAFLD. I have no further comments.

Considering that changing table number one would cause a significant change in the results section of the article, we decided to make this suggestion of the esteemed reviewer in the form of supplementary tables number one and two.
